# Impact of negative links on the structural balance of brain functional network during emotion processing

**DOI:** 10.1038/s41598-023-43178-8

**Published:** 2023-09-25

**Authors:** Farhad Soleymani, Reza Khosrowabadi, Mir Mohsen Pedram, Javad Hatami

**Affiliations:** 1 Institute for Cognitive Science Studies, Tehran, Iran; 2 Institute for Cognitive and Brain Science, Shahid Beheshti University GC, Tehran, Iran; 3 Faculty of Engineering, Kharazmi University, Tehran, Iran; 4 Faculty of Psychology and Educational Sciences, University of Tehran, Tehran, Iran

**Keywords:** Cognitive neuroscience, Emotion, Network models

## Abstract

Activation of specific brain areas and synchrony between them has a major role in process of emotions. Nevertheless, impact of anti-synchrony (negative links) in this process still requires to be understood. In this study, we hypothesized that quantity and topology of negative links could influence a network stability by changing of quality of its triadic associations. Therefore, a group of healthy participants were exposed to pleasant and unpleasant images while their brain responses were recorded. Subsequently, functional connectivity networks were estimated and quantity of negative links, balanced and imbalanced triads, tendency to make negative hubs, and balance energy levels of two conditions were compared. The findings indicated that perception of pleasant stimuli was associated with higher amount of negative links with a lower tendency to make a hub in theta band; while the opposite scenario was observed in beta band. It was accompanied with smaller number of imbalanced triads and more stable network in theta band, and smaller number of balanced triads and less stable network in beta band. The findings highlighted that inter regional communications require less changes to receive new information from unpleasant stimuli, although by decrement in beta band stability prepares the network for the upcoming events.

## Introduction

Emotion processing is an undeniable part of human life and several theories have been proposed that how emotional contents of stimuli are perceived^1–3^. For instance, categorical theory explains the perceptual mechanism based on the basic emotions such as anger, disgust, fear, happy, sadness, contempt, and surprise proposed by Paul Ekman^4^. In this theory, the complex emotional states are defined as a combination of these basic emotions^5–8^. Nevertheless, there is no convergence about the number of basic emotions in different research^9–11^. Therefore, a dimensional approach which defines the emotions based on the pleasantness, arousal and dominance levels was proposed by James Russell^12^. In this approach, the basic and complex emotional states are similarly defined based on the arousal, valence and dominance levels^13,14^.

It is believed that perception of emotion is associated with physiological changes^15,16^. Among the physiological changes, neurophysiological signals have gained of interest in the recent years^17–19^. In fact, various features could be extracted from the brain activities to point the perceived emotional state^20–22^. Literatures have pointed that hemispheric laterality has a major role in perception of emotions^9,23–25^. In addition, emotion processing is performed in a frequency specific manner^1,9,24–26,26–28^. It has been reported that lower band frequencies specially theta waves (4–8 Hz) and higher frequencies mainly in beta waves (13–30 Hz) are important in perception of valence level of emotional stimuli^11,29–32^. For instance, it has been shown that unpleasant stimuli significantly increase activities at the theta frequency band in frontal, parietal, occipital, and central-occipital electrodes^33^. In addition, asymmetric activities at theta, alpha (8–13 Hz), beta and gamma (> 30 Hz) frequency bands in frontal areas have been observed during pleasant and unpleasant stimuli^31,32,34,35,35–37^. Whereas, higher activities in right hemisphere during negative events, and more activated left hemisphere during positive events have been reported^14,17,38–45^. Moreover, activities at the midline areas between the two hemispheres have been pointed as a significant marker for emotional perception. For instance, alteration of midline theta power for negative emotion^41^, increase of theta power in middle frontal area during pleasant stimuli^46^, and changes of midline alpha and beta power for positive emotion have been stated^40,47^. In addition to laterality, anti-synchrony between heterologous regions such as anti-synchrony between prefrontal and posterior areas at theta and beta frequency bands have been described^48^.

Although emotion processing is associated with activities at some particular brain areas such as amygdala, but it is believed that synchronized brain circuits must be responsible for emotion processing^49^. Therefore, synchronization between activities of the brain areas known as functional connectome has also been investigated for discrimination of emotions. Among the synchronization methods, phase synchrony seems to be more reliable and could compensate the volume conductance effect^50^. Hence, synchronization of phase index of EEG signals has been investigated during the emotion perception. Studies have shown that phase synchrony (functional link) between regional brain activities is different in various emotional states that could be used for discrimination of them from each other^51,52^. In this study we wanted to look at the brain functional network stability in different emotional states, therefore, a consideration about organization of positive and negative was required^53–56^. Since the range of phase synchrony is between 0 to 1, deviation from the average values of the links while exposed to neural images were calculated Zscore = (FC_ps_emotional state_-mean(FC_ps_neutral state)_))/std(FC_ps_neutral state)_). So, negative values were also obtained and entitled as the negative links or anti-synchronies in the networks of emotional states.

In this regard, importance of phase synchrony for perception of unpleasant stimuli^57^, and discrimination of pleasant and unpleasant stimuli have been stated^29,58,59^. Nevertheless, the important role of anti-synchrony has been poorly understood. The anti-correlated activities described by Fox et al.^60,61^ could help us to understand impact of negative links. In this study, we hypothesized that perception of the emotions could be linked to the number and quality of anti-correlations (negative links). Subsequently such a functional topology could influence the network behavior and put a tag of pleasant or unpleasant on the stimuli.

Functional topology of the links could be investigated by the structural balance theory^53,54^ which is dependent to the quality of triadic associations (associations of three connections)^26^. The balance energy level of a network describe the network stability^55^, and how negative links could provide the requirements of a change for a frustrated network and lead to the behavioral changes.

There have been extensive studies on the processing of emotional states in the brain. However, these studies have not measured the stability of different emotional states on the brain's functional network. This study proposes a novel approach by considering the structural balance theory and balance energy. Balance theory refers to the social structural balance theory introduced by (Heider 1946). Structural balance theory explains the behavior of two entities (people) in the presence of the third part (people or objects). Consequently, we consider the brain nodes (EEG channel) as the entities to consider the behavior of two pair channels in the presence of the third channel. Balance energy which was introduced by (Marvel et al. 2009), explains that interaction between more than two objects can produce energy(Hamilton form). Therefore, this possibility has arisen to measure the level of stability in different emotional states by measuring the level of balance energy.

In this study, we investigated how number and organization of the negative links are different while pleasant and unpleasant stimuli are perceived. In addition, we tried to show how this organization with their tendency to make negative hubs influence the structural balance of the network in pleasant and unpleasant emotional states.

## Results

**Figure 1:**
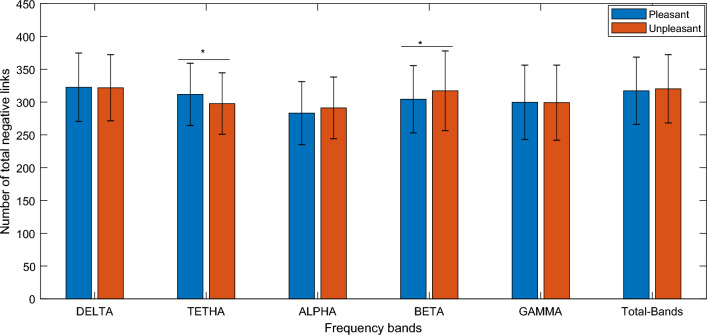
Comparison between quantity of negative links in pleasant and unpleasant stimuli. *Denotes a significant difference with *p* value < 0.05. In the bar plot, average and standard errors of the all subjects’ values have been presented. The error bar represents the standard deviation of the feature distribution.

In this study perception of emotional contents of images was experimentally investigated using the EEG signals acquired during exposure to the stimuli. After preprocessing of the EEG data, the connectivity matrix was formed based on the characteristics of phase locking values between pairs of electrodes, and then the signed network was formed by calculating the z scores (considering mean and standard deviation from the responses to neutral images). Subsequently, number of negative links for the pleasant (high valence) and unpleasant (low valance) were statistically compared. Significant differences between pleasant and unpleasant network were observed in Theta (T value = 3.3134, *p* value = 0.0011) and Beta (T value = − 2.4954, *p* value = 0.0133) frequency bands (see Fig. 1). It indicates that pleasant network has a higher amount of negative links in theta band and lower amount in beta band as compared to the unpleasant network. The legend presents the type of stimuli. The pleasant was introduced as high valence stimuli and the unpleasant as low valence stimuli. Representations in the box-plot format are also provided in the Supplementary Materials.

**Figure 2:**
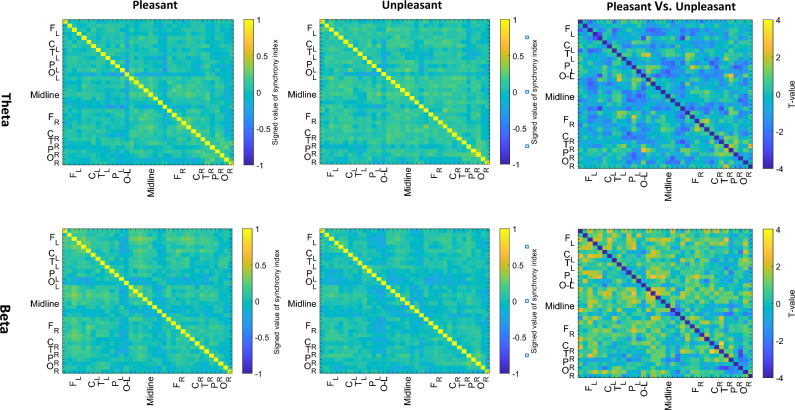
Comparison of brain functional connectivity networks during the process of pleasant and unpleasant stimuli. The first and second columns present group average of the PLVs and the third column presents t value of paired wised comparison of the pleasant and unpleasant conditions.

In addition, connection-wise average of signed networks in pleasant and unpleasant conditions were also statistically compared to highlight the significant connections (In Fig. 2 the arrangement of the EEG channels consists of left frontal: Fp1-F7-F5-F3-F1, left central: FC3-C3, left temporal: FT7-T7-TP7, left parietal: CP3-P7-P3, left occipital: O1, midline: Fpz-AFz-Fz-FCz-Cz-CPz-Pz-Oz and right frontal: Fp2-F2-F4-F6-F8, right central: FC4-C4, right temporal: FT8-T8-TP8, right parietal: CP4-P4-P8 and right occipital: O2). Significant results were observed in functional links between left frontal and left parietal regions with the midline areas and right central and right occipital regions with left parietal areas in theta band. Moreover, functional connectivity between central and temporal regions with parietal areas at the right hemisphere in beta frequency band were substantially differentiable in pleasant and unpleasant stimuli.

**Figure 3:**
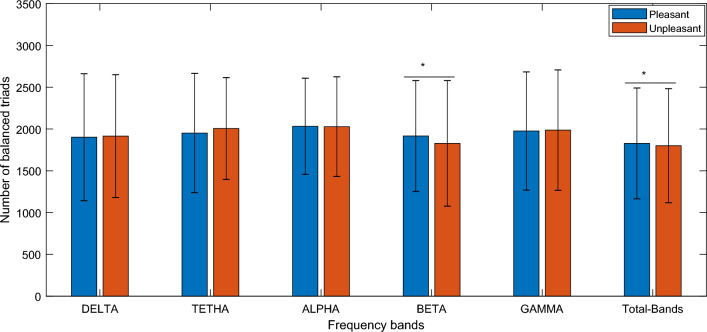
Comparison of quantity of balanced triads in pleasant and unpleasant states (summation of T1, and T3). T1 denotes triads with one positive link and T3 presents triads with three positive links. *Denotes a significant difference with *p* value < 0.05. In the bar plot, average and standard errors of the all-subjects’ values have been presented.

Then, all the possible triads combination was calculated from the signed matrix without repetition. Subsequently, number of balance and imbalanced triads based on the number of positive links (T0–T3) were calculated. Statistical comparison of pleasant and unpleasant networks showed balanced triads (summation of T1 and T3) were significant higher in the pleasant network at the beta frequency band (T value = 2.431, *p* value = 0.0154) see Fig. 3.

**Figure 4:**
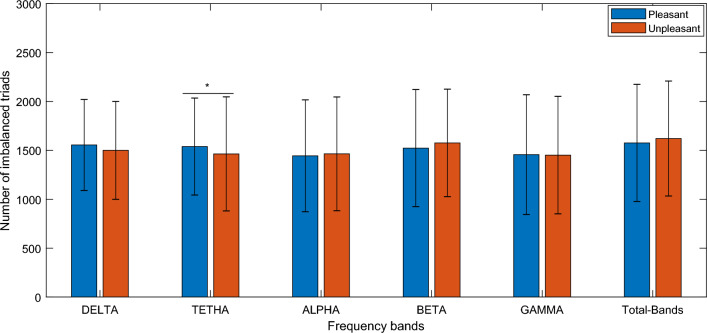
Comparison of quantity of imbalanced triads in pleasant and unpleasant states (summation of T0, and T2). *Denotes a significant difference with *p* value < 0.05. Pleasant denotes high valence stimuli and unpleasant denotes low valence stimuli. In the bar plot, average and standard errors of the all subjects’ values have been presented. T0 presents no positive link in triad association and T2 presents two positive link in triad association.

In contrast, number of imbalanced triads (summation of T0 and T2) were significantly higher in pleasant condition at the theta frequency band (T value = 3.0701, *p* value = 0.0023) see Fig. 4.

**Figure 5:**
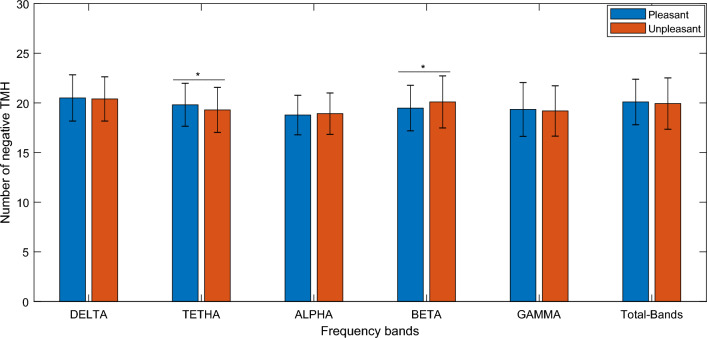
Comparison of tendency of negative links to make a hub in networks of pleasant and unpleasant stimuli.*Denotes a significant difference with *p* value < 0.05. Pleasant denotes high valence stimuli and unpleasant denotes low valence stimuli. In the bar plot, average and standard errors of the all subjects’ values have been presented.

Subsequently, tendency of negative links to make a hub were compared in brain functional networks of pleasant and unpleasant conditions. The result showed that negative links have more tendency to make a hub (higher TMH) in the network of pleasant condition at the theta frequency band (T value = 2.6565, *p* value = 0.0085) and lower tendency (smaller TMH) in beta frequency band (T value = − 2.7217, *p* value = 0.0070) see Fig. 5.

**Figure 6:**
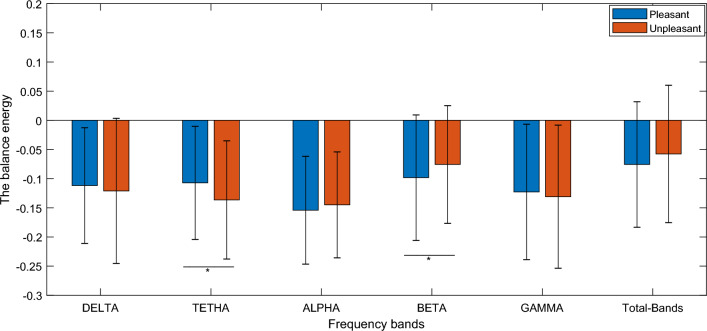
Comparison of balance energy level (stability) of pleasant and unpleasant stimuli. *Denotes a significant difference with *p* value < 0.05. Pleasant denotes high valence stimuli and unpleasant denotes low valence stimuli. In the bar plot, average and standard errors of the all subjects’ values have been presented.

Adding up the balanced and the imbalanced triads, analysis of balance energy level of the networks of pleasant and unpleasant stimuli were also performed to compare the stability of the networks in two conditions. The results pointed that the brain functional networks of pleasant stimuli are significant less stable (higher balance energy level) in theta frequency band (T value = 3.1262, *p* value = 0.0020) and more stable (lower balance energy level) in beta frequency band (T value = − 2.2517, *p* value = 0.0253) see Fig. 6.

## Discussion

In this study, we aimed to differentiate stability of brain functional networks in pleasant and unpleasant emotional states. We compared the quantity and the topology of negative links and the quality of their triadic associations during process of pleasant and unpleasant image stimuli. Subsequently, balance energy level which could describe the networks stability were compared in the above mentioned conditions. A group of healthy participants were recruited and their electro-cortical signals were recorded while they were exposed to pleasant and unpleasant images from IAPS dataset. Comparison of functional signed networks denoted that percentage of negative links in functional connectome of pleasant stimuli is higher than unpleasant stimuli in the theta band frequencies, while it is lower in beta band frequencies. Moreover, the negative links have a lower tendency to make a hub in the theta band, while this tendency is increased in beta frequencies. Such an organization decreases number of imbalanced triads and brings the network to a more stable condition in theta band, and increases the number of balanced triads and lead the network to a less stable state in the beta band. Importance of theta and beta waves in process of pleasantness.

In fact, emotions are processed in the brain by convergence and divergence of brain waves and synchronization of rhythmic brain circuit related to the emotional state. It has been reported that lower band frequencies specially theta waves (4–8 Hz) and higher frequencies mainly in beta waves (13–30 Hz) are important in perception of valence level of emotional stimuli^11,29–32^. For instance, it has been shown that unpleasant stimuli substantially increase activities at the theta frequency band in frontal, parietal, occipital, and central-occipital electrodes^33^. The theta waves have been associated with attention and by inhibition of automatic responses and they could influence the computation in short-term memory^62^ which is required for cognitive control^50^. For instance, the theta waves at the frontal midline area could use a synchronization policy to communicate with other brain areas as the cognitive control mechanism for intentional process of emotions. We think our findings on synchronized circuit between the mid-frontal and the left frontal and parietal regions also in the same line present a similar understanding. In process of pleasant stimuli, attention to the stimuli is intentionally persuaded and inter regional communication is enhanced. In contrast, the unpleasant stimuli may not need to be attended and do not require to change the inter regional communications.

On the other hand, the beta band activities are involved in logical processing of the events and could direct the attentional mechanism to the cognitive task and prepare the brain network for upcoming events. Therefore, these brain activities could play a predictor role for perceiving the contents of the stimuli and prepare the motor responses. Such a mechanism is a need when one faces to a negative/unpleasant stimulus. In addition, the differential amount of negative links was mostly observed in the networks of theta and beta frequency bands. These findings are compatible with the previous studies on brain connectivity during emotion processing that have shown the emergence of the theta frequency band^21–24,26,46^, and changes in beta waves^23,24,38,43,44^ during low/high levels of valence.

Nevertheless, current sample size, age range and gender of participants are among the limitations of this study. Moreover, emotional stimuli must be considered from various media including face, voice, music and video as well. Furthermore, emotional perception was measured using a self-report scale and thinking of other possible methods could be beneficial.

## Conclusion and future directions

In summation, we think during process of pleasant contents inter regional communications must be enhanced to better attend the stimuli. This will cause an increase in theta waves and will change its network accordingly. In contrast, during the unpleasant stimuli, the related functional network may not require to remain in the same state, therefore, do not need to receive new information from the unpleasant stimuli anymore. Therefore, the functional brain network requires to be changed accordingly and be prepared for future events, which is performed by decrement in beta band stability. Therefore, investigation of dynamic connectivity networks while processing a predefined trend of emotional stimuli seems a horizon for future works.

## Methods

### Participants

Thirty people participated in this study. Among them, ten candidates were excluded because of their scores in general healthcare questionnaire (GHQ) and a questionnaire for measuring their long-term depression, anxiety and stress score (DASS-21). In addition, EEG data of two subjects were still noisy in some trials (even after preprocessing); Therefore, we decided to exclude their data as well. So, eighteen subjects with 30 trials (6 for each of 5 emotional states) of clean EEG data were included in this study. All the participants were male, right handed and age ranged between 18 and 35 years old with no history of neurological or neuropsychiatric disorders and with no addiction background. All the participants filled and signed a written and informed consent form (for both study participation AND publication of identifying information/images in an online open-access publication) prior to the experiment and answered to the general health questioner. Experiment was conducted in accordance with the 1964 Helsinki federation and the ethical review board of the Shahid Beheshti University approved the ethics of the experiment with reference number IR.SBU.REC.1400.195. In addition, participants were monitored for long term stress, anxiety, and depression by DASS 21 scale^63^ questionnaire and participants with score above 4 in depression, 3 in anxiety, and 7 in stress domains were excluded.

### Stimuli images selected from the international affective picture system (IAPS)

The IAPS is a widely used database of categorized images to study emotions and attention. The basic feature of the IAPS is a detailed list of the average ratings for arousal, valance, and dominance levels of each picture. The normative rating for the IAPS Dataset has been performed using a graphical rating scale named as self-assessment model proposed by James Lang^64^. The Self-Assessment Manikin (SAM) questionnaire is a non-verbal visual self-expression method that measures criteria such as valence, arousal and dominance related to the emotional response of people. The SAM could be implemented as an interactive computer program or using a pen-and-paper version. In the present study, a SAM with a 9-point scale was used. For assessment of valence level, 1 was considered as the least intensity of pleasantness and 9 for the highest level of pleasantness of the stimulus^65^. The rating was performed after presenting each image by pressing the related number on the keyboard.

### Experimental procedure

**Figure 7:**
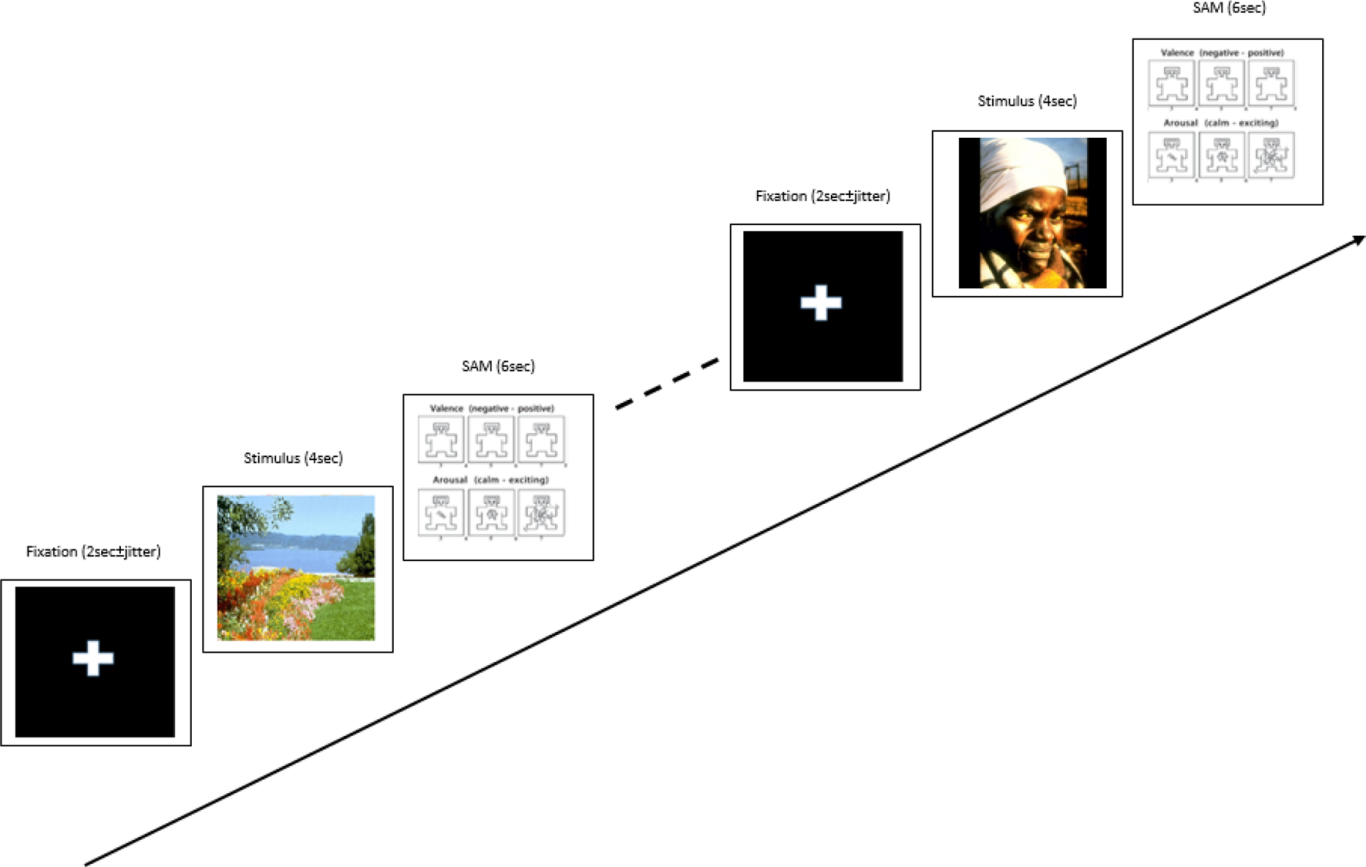
Stimuli presentation paradigm.

In this study, participants were exposed to five categories of emotional images. The paradigm of stimuli presentation is presented in Fig. 7. 30 stimuli images were selected from IAPS dataset that included six images in categories of (a) high valence (> 6) and low arousal (< 3), (b) high valence (> 6) and high arousal (> 6), (c) low valence (< 3) and low arousal (< 3) (d) low valence (< 3) and high arousal (> 6), € neutral images. The high valence categories were considered as pleasant and low valence categories were considered as unpleasant stimuli. The stimuli presentation was programmed in MATLAB software version 2019 using the psychtoolbox functions.

The participants were sited on a comfort chair in front of 19-inch screen placed 75 cm away of the subjects. The experimental procedure was explained to the participants and an announcement about their readiness was acquired by pressing the space key. After pressing the space key, a fixation sign "+" was appeared on the screen for (2 ± 0.2 s) to avoid prediction of stimuli times by jittering and prevent the task adaptation. Then, the image stimulus was presented for 4 s while participant was only watched the stimulus. It should be mentioned that images were randomly presented but counterbalanced for each category (6 images for each of five emotional states). After presentation of each stimulus, participant emotional feeling of the image was measured by the self-assessment manikin (SAM) questionnaire. The SAM was presented using a 9-point Likert scale for arousal and valance levels separately, and the participant was asked to rate the perceived stimulus in terms of arousal or valance in a period of 6 s. This process continued until all stimuli were presented. In addition, brain responses of the participants were recorded using a 36-channel EEG amplifier designed by the Liv Intelligent Technology Ltd co. (www.lliivv.com/) while they were exposed to the pictorial stimuli. The EEG data recording was performed by sampling frequency of 256 Hz and impedances of electrodes were checked to be below 5 kHz. In addition, 4 min of resting state EEG data (2 min in Eyes-close, and 2 min in eyes-open conditions) were also recorded prior to the stimuli presentation. The reference was average of activities of electrodes placed behind the ears and presentation and recording systems were synchronized through sending trigger markers on the parallel port.

### Data preprocessing

**Figure 8:**
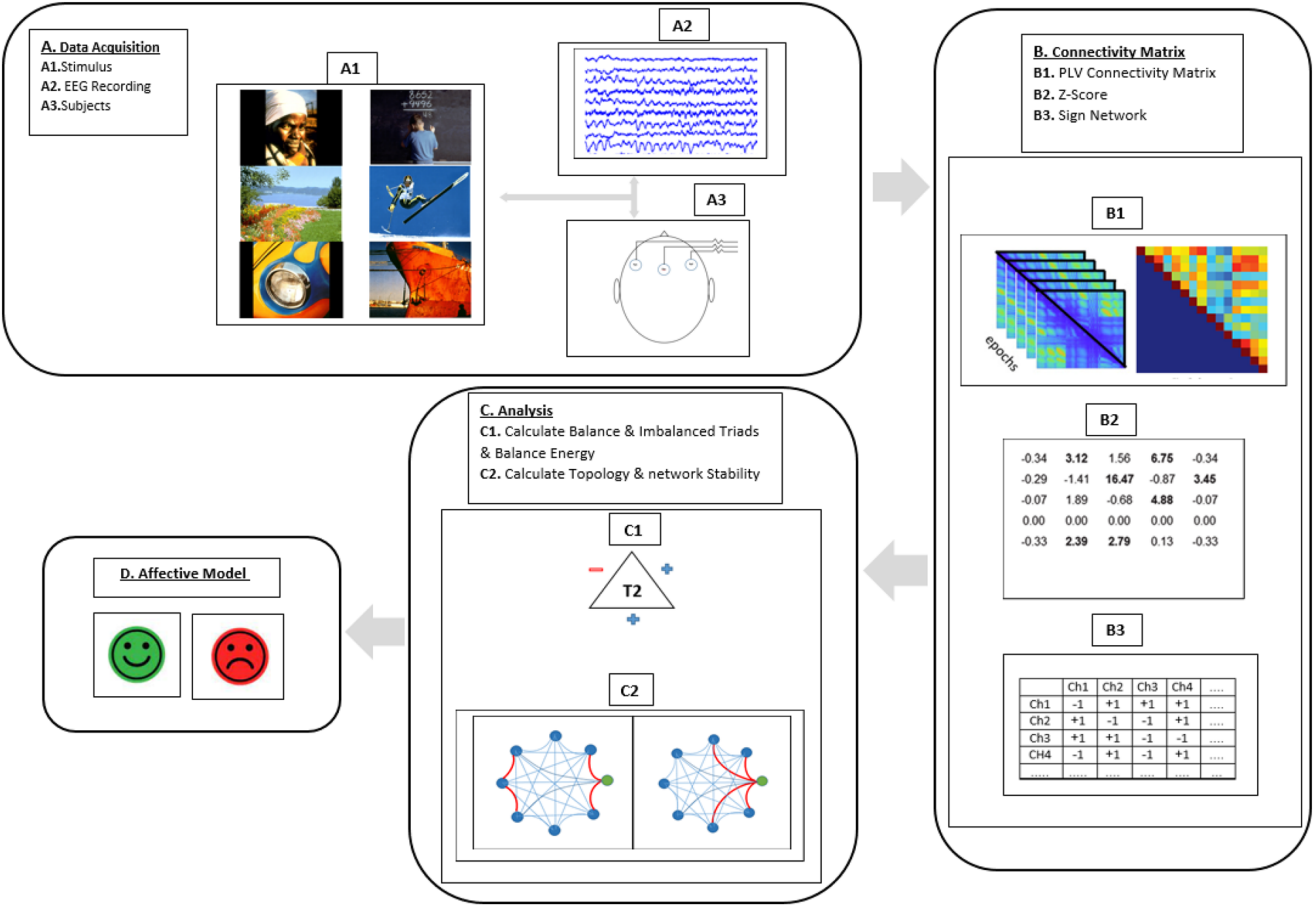
Schematic of data gathering and analysis. (**A**) Behavioral and EEG data gathering. (**B**) Feature extraction of EEG signals: preprocessing, extraction of functional connectivity network, forming the sign network. (**C**) Counting of negative links, balance and imbalance triads, negative TMH and balance energy level of the network. (**D**) Statistical comparison of the pleasant and unpleasant networks.

A standard pre-processing was performed on the recorded EEG data using the eeglab toolbox^66^. For this purpose, recorded data were band-pass filtered in the range of 0.5–45 Hz using an FIR filter. Then, using ICA method, independent sources were preserved and noise sources were eliminated. In addition, after visual observation of the data, noisy trials were removed. The preprocessed data of all the participants categorized according to valence dimension and implied for further processing. A schematic representation of data gathering procedure and analysis of behavioral and EEG data is shown in Fig. 8.

### Extraction of functional connectivity network using the phase lock value (PLV)

Association between activities of different EEG electrodes can be calculated based on their amplitude or phase synchrony. It has been reported that phase synchrony based methods are more reliable in terms of test–retest reliability^67^. Therefore, the phase locking approach suggested by Lachaux (1999) was implied in this study. This criterion is considered as an estimator of the relationship between two signals (binary relationships between brain signals)^68^. The PLV was calculated for EEG of pleasant, unpleasant, and neutral stimuli at Delta (0.5–4 Hz), Theta (4–8 Hz), Alpha (8–13 Hz), Beta (13–30 Hz), lower Gamma band (30–45 Hz) and the total frequency band (0.5–45 Hz) using the HERMES toolbox^69^. Subsequently, Z-Scores of the PLV values were calculated using Eq. (1).

1 $$Z_{ij} = \left( {PLV_{ij} - \mu_{ij} } \right)/SD_{ij}$$

where PLV_ij_ denotes the phase locking value between EEG of channel i and channel j during the pleasant/unpleasant stimulus, µ_ij_ and SD_ij_ represent average and standard deviation of the PLVs between channel i,j during the neutral stimuli. After that, the signed networks were computed by applying sign function on the z scores.

### Structural balance network

Balance theory deals with people's attitudes towards issues, which was first proposed by Fritz-Heider. The theory states that the attitude of people in social issues influenced and changed in relation to others. Modeling the concept of balance expressed by examining a triple relationship, which elements of this triangle are combination of people, others and subjects. People and others have positive or negative attitudes towards different issues. The attitude of people under the influence of other people's opinions changes depending on the type of relationship, be it friend or enemy^53,70,71^.

### Possible combinations of triads

If we consider the relationship between nodes as signed, then we will have signed triads. If in a social network, we consider nodes as people, in that case, the sign of communication indicates the relationships between them by friendship and enmity. In this case we have four types of triads (strong balanced triad including three positive relationships T3 = +++ , weak imbalanced triad including two positive relationships T2 =−+ + , weak balanced triads including a positive relationship T1 =− + strong imbalanced triads without any positive relationship T0 =– – –)^54^.

### Balance energy level

In the definition of energy, connections between three or more nodes take on the concept of energy. The lower the energy of these connections, the higher the stability of the system, and on the other hand, if the tendency of these connections is towards instability (higher energy), they have the ability to change the behavior of the system. In fact, the less energy needs to be changed. As a result, to evaluate the level of stability, if a network place at a lower minimum level in terms of energy level, it will be more stable. Marvel et al. (2009) proposed the relationship in the form of network balance. In that relationship, the energy of a triads is calculated by considering the relationship of friendship as + 1 and the relationship of enmity as − 1^43,72^.

Total balance energy was calculated using Eq. (2). In Eq. (2), n represents the number of possible triads combination and S_ijk_ represent the sign of ijk nodes in the form of triads association.

2 $$U = - \frac{1}{\left(\frac{n}{3}\right)} {\sum }_{\frac{i,j,k}{i\ne j\ne k}}\,{ S}_{ij}\, {S}_{ik}\, {S}_{jk}$$

### Tendency to make hub (TMH)

Each network consists of nodes and each node connected with other nodes according to the brain functional paradigm. Nodes that have a higher degree of connections with other nodes called central nodes or hubs. For the tendency of a network's connections to create a hub, a criterion called the tendency to make hub (TMH) has been defined. TMH was also calculated using Eq. (3). In Eq. (3), the N represents the number of nodes (number of EEG electrodes) and D_i_ represent the nodes’ degree.

3 $$TMH= \frac{{\sum }_{i=1}^{N} {D}_{i}^{2}}{{\sum }_{i=1}^{N} {D}_{i}}$$

Positive TMH intended to calculate the degree of tendency to make hub using positive connections in the network, and similarly, negative TMH intended to calculate the degree of tendency to form a hub using negative connections. Effect of TMH on the stability of the network has been presented in previous Seberi et al. works, Fig. 5^53^.

### Statistical analysis

The extracted features were checked for the normality using the kolmogrov-smironov test. Considering the normality of the data, a paired-wise t-test was used to measure the substantial difference between the pleasant and unpleasant conditions. First of all, the number of negative links were statistically compared. After, number of balanced and imbalanced triads were also compared and tendency of negative links to make hubs were also compared in the two mentioned conditions. The similar procedure was performed for each frequency band separately. Therefore, the final results were corrected for multiple comparison effect using the Bonferroni method and *p* value < 0.05, FWE corrected was considered to point the significant level.

### Ethical approval

All the participants filled and signed a written and informed consent form prior to the experiment and answered to the general health questioner. Experiment was conducted in accordance with the 1964 Helsinki federation and the ethics of the experiment was approved by the research ethics committees of the Shahid Beheshti University with reference number IR.SBU.REC.1400.195.

### Supplementary Information

Supplementary Figures.

## Data Availability

The Functional connectivity matrixes (PLV values for each image) of all the participants are publicly available on “m9.figshare.22256803/10.6084”. The raw data are however available from the authors upon reasonable request and with permission of the corresponding author.
