# Supplementary material for: Impact of negative links on the structural balance of brain functional network during emotion processing
Source: Sci Rep. 2023 Sep 25;13:15983. doi: 10.1038/s41598-023-43178-8 (PMC10519959; doi:10.1038/s41598-023-43178-8)
Supplement: Supplementary file 1 — Supplementary Figures. [file 41598_2023_43178_MOESM1_ESM.docx]

**Title: Impact of negative links on the structural balance of brain functional network during emotion processing**

**Supplementary Materials**

**Figure S1.** Comparison between quantity of negative links in pleasant and unpleasant stimuli

**Figure S2.** Comparison of quantity of balanced triads in pleasant and unpleasant states (summation of T1, and T3).

**Figure S3.** Comparison of quantity of imbalanced triads in pleasant and unpleasant states (summation of T0, and T2).

**Figure S4.** Comparison of tendency of negative links to make a hub in networks of pleasant and unpleasant stimuli

**Figure S5.** Comparison of balance energy level (stability) of pleasant and unpleasant stimuli.

|  | **Pleasant Vs. Unpleasant positive links** | **Pleasant Vs. Unpleasant negative links** |
| --- | --- | --- |
| **Theta** | **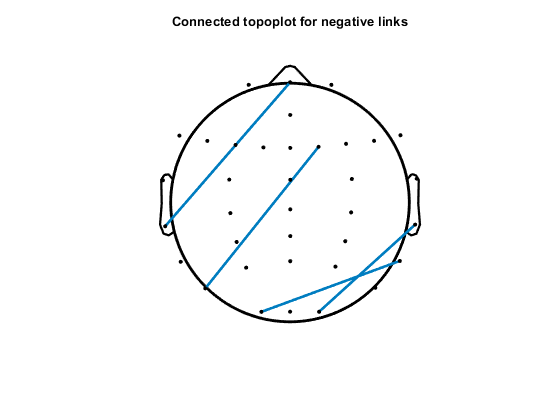** | **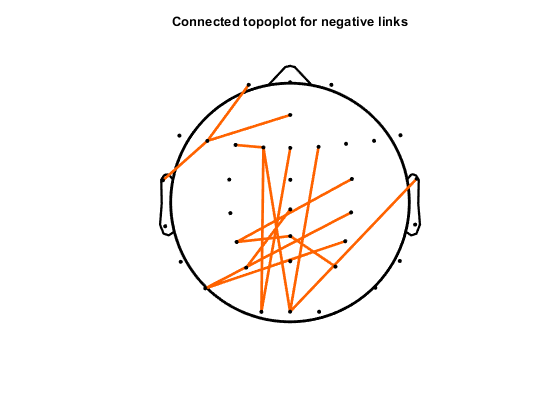** |
| **Bata** | **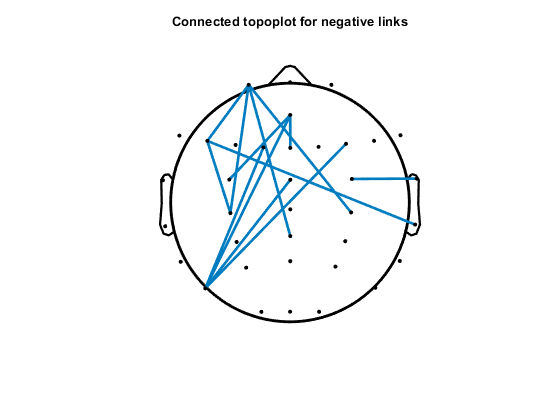** | **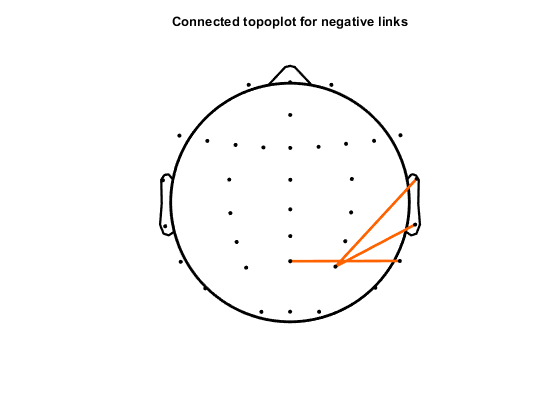** |

**Figure S6.** Comparison of brain functional connectivity networks during the process of pleasant and unpleasant stimuli. The first and second columns present group average of the PLVs and the third column present t value of paired wised comparison of the pleasant and unpleasant conditions.

**The following figures present the result of this study using PLI features**

**Figure S7.** Comparison between quantity of negative links in pleasant and unpleasant stimuli

**Figure S8.** Comparison of quantity of balanced triads in pleasant and unpleasant states (summation of T1, and T3)

**Figure S9.** Comparison of quantity of imbalanced triads in pleasant and unpleasant states (summation of T0, and T2)

**Figure S10.** Comparison of tendency of negative links to make a hub in networks of pleasant and unpleasant stimuli

**Figure S11.** Comparison of balance energy level (stability) of pleasant and unpleasant stimuli

**Figure S12.** Comparison between quantity of negative links in pleasant and unpleasant stimuli

**Figure S13.** Comparison of quantity of balanced triads in pleasant and unpleasant states (summation of T1, and T3)

**Figure S14.** Comparison of quantity of imbalanced triads in pleasant and unpleasant states (summation of T0, and T2)

**Figure S15.** Comparison of tendency of negative links to make a hub in networks of pleasant and unpleasant stimuli

**Figure S16.** Comparison of balance energy level (stability) of pleasant and unpleasant stimuli
